# Long-term impact of olfactory dysfunction on daily life

**DOI:** 10.1007/s00508-020-01751-5

**Published:** 2020-10-21

**Authors:** Alice B. Auinger, Gerold Besser, David T. Liu, Bertold Renner, Christian A. Mueller

**Affiliations:** 1 Department of Otorhinolaryngology and Head and Neck Surgery, Medical University of Vienna, Waehringer Guertel 18–20, 1090 Vienna, Austria; 2 Institute of Experimental and Clinical Pharmacology and Toxicology, University of Erlangen-Nürnberg, Erlangen, Germany; 3 Institute of Clinical Pharmacology, Medical Faculty Carl Gustav Carus, Technical University Dresden, Dresden, Germany

**Keywords:** Anosmia, Hyposmia, Quality of life, Prognosis, Smell

## Abstract

**Background:**

Olfactory dysfunction (OD) is common in the general population, affects the quality of life (QoL), and is suspected to cause depression. Long-term outcome data are lacking and there is a need to improve patient counselling regarding prognosis. We aimed to assess subjective long-term recovery rates, the QoL, and mood disturbance in a group of 65 patients, who were affected with OD.

**Methods:**

Out of 325 patients treated for OD between 2003 and 2009  at a smell and taste clinic, 65 patients were included for a follow-up after an average of 8.6 years. A total of 28 patients answered questionnaires only and 37 patients were provided with an additional smell identification test. Among others, questionnaires included a short form of the World Health Organization quality of life questionnaire (WHOQOL-BREF) and the Beck’s depression inventory.

**Results:**

In the long run, subjective improvement was stated in 33.8% of all patients, with the highest rate of 42.3% in patients with postinfectious OD. The subjective rating of olfactory function on a visual analogue scale was significantly higher at study follow-up compared to first clinical contact (median 1.25 vs. 4.5; U = 469.5, *p* = 0.001), as were mean identification scores (6.0 ± 3.0 vs. 8.0 ± 4.0, t(18) = 2.51, *p* = 0.021). The QoL in general was considered reduced in 40% of all patients at follow-up. Furthermore, participants exhibited only minor, if any, depressive symptoms.

**Conclusion:**

Despite negative effects of OD on certain activities in daily life, such as cooking, detecting spoiled food, or personal hygiene, it seems that the patients included in this study adapted to the OD in the long-term. The current findings should aid clinicians in patient counselling.

**Electronic supplementary material:**

The online version of this article (10.1007/s00508-020-01751-5) contains supplementary material, which is available to authorized users.

## Introduction

Olfactory dysfunction (OD) is common and affects up to one quarter of the general population, with even higher prevalence rates in the older generation [1–3]. Most common causes of OD include upper airway infection (18–45%), sinonasal disease (7–56%), head trauma (8–20%), toxic exposure (2–6%), and congenital defects/disorders (0–4%) [4]. Although the olfactory system is remarkably plastic and harbors neural stem cells over a lifetime, regeneration can be inefficient and therapeutic options are limited depending on the cause of OD. In terms of postinfectious OD, short-term recovery rates of 6–8% within 4 months [5], 21–35% within 1 year [6, 7] and 67% after 37 months [8] have been reported. In contrast, only 10–20% of patients with posttraumatic OD experience an improvement [9]. Traumatic OD is caused either by a blocked nasal passage, direct trauma to the olfactory nerve or by hemorrhage and/or contusion within the central nervous system [10]. Due to an initial unawareness of OD after traumatic brain injury, diagnosis and appropriate counselling can be delayed [11, 12]. A considerable proportion of people struggle with the consequences of OD but studies evaluating long-term recovery rates are surprisingly lacking.

In particular, studies indicate that patients suffering from smell disorders are negatively affected in their general quality of life (QoL) [13]. This reduction might be explained by several restrictions in daily life activities. Impaired retronasal olfactory function leads to decreased flavor perception and less food enjoyment in up to 69% [14, 15], which can result in reduced appetite in up to one third or even 56% of affected patients [14, 15]. About 3–20% of patients report eating more and 20–36% less resulting in gaining or losing weight, respectively [16]. Another problem reported by patients with OD is difficulties with cooking in up to 73% and detecting spoiled food [14, 17].

The risk for accidents increases as OD can lead to the inability of detecting fire, gas or smoke in up to 61% [15, 17–20]. One of the most negative effects of OD is an impaired ability to perceive own body odors resulting in social insecurity [14, 15]. Patients with OD might become unable to work as cooks, wine makers, nurses, and other professions that are dependent on olfactory function. These effects of OD can contribute to a reduction in general QoL, and patients with OD are more often prone to depression but it has been a matter of discussion whether depression is caused by OD or vice versa [21, 22].

Whether these associations are consistent in the long term, even after treatment for OD or if patients adapt to their dysfunction and show reduced general QoL is of interest. Most importantly, the individual ability to deal with OD might be more important for the patient than the olfactory function per se. In a study conducted by Landis et al. [23] the majority of patients with OD reported receiving poor information about diagnosis and prognosis. Therefore, in order to improve patient counselling, this work was carried out to assess long-term data of subjective recovery rates in a group of patients who were treated for chemosensory disorders at our specialized taste and smell clinic.

## Methods

### Participants

A total of 325 patients suffering from chemosensory dysfunction who attended the smell and taste clinic at the Medical University Hospital of Vienna between 2003 and 2009, were screened for this study. We retrospectively reviewed the patients’ charts to obtain demographic data on visiting patients. Between 2003 and 2009, at the first consultation all patients underwent clinical examinations of the ear, nose and throat and further olfactory testing was performed in selected cases in a screening fashion [24, 25]. From this examination the following parameters were used for this study: cause and duration of OD, olfactory function by means of the identification smell test, self-rated smell function by means of a visual analogue scale (VAS 0 = no function, 10 = best function) and treatment. At first consultation, patients were routinely asked to return for a clinical follow-up 6 months later (referred to as short-term follow-up).

Between 2014 and 2016, all 325 screened patients were invited to take part in a clinical follow-up examination and questionnaires were sent to them. Patients who did not respond were contacted by telephone and asked for a clinical follow-up. Out of all invited patients 65 (27 males, 38 females, mean age ± standard deviation 64.0 ± 16.5 years) were included in the study (response rate 20.0%); 37 (11.4%) responded to the invitation for a clinical follow-up appointment and 28 (8.6%) returned questionnaires but were not able to take up the invitation. The questionnaires included the following parameters: negative/positive effects of OD, impact of OD on QoL, self-rated smell function on a VAS, a short form of the World Health Organization quality of life questionnaire (WHOQOL-BREF, the WHOQOL group, 1998), and the Beck’s depression inventory II (BDI-II) [26–28]. As QoL and mood questionnaires were not routinely assessed at the first clinical contact, changes could not be detected at follow-up and parameters were therefore collected at a single time point. Due to the availability, we chose general, non-olfaction-specific questionnaires. Patients who presented for a follow-up examination were additionally provided with a clinical examination of the ear, nose and throat and an identification smell test.

**Fig. 1:**
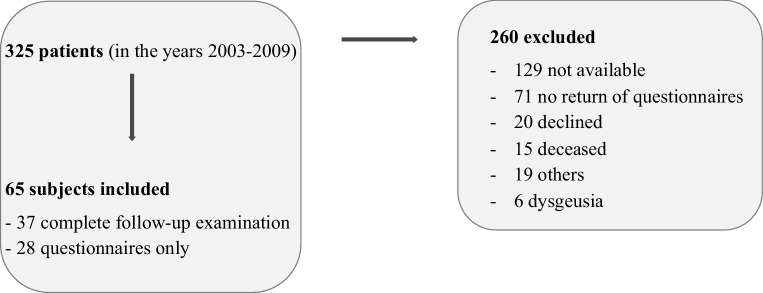
Invited patients and reasons for exclusion

The mean follow-up was 8.6 ± 1.9 years (range 5–16 years). Fig. 1 depicts invited patients and the reasons for exclusion. As only six patients suffered from dysgeusia (1.8%), we excluded them from further analysis. Initial treatment consisted of oral steroids (25 mg prednisone for 10 days) in most of included patients (*n* = 51, 78.5%). Septoplasty was performed in one (1.5%) patient, and functional endoscopic sinus surgery in another (1.5%) patient.

The study protocol was approved by the local ethics committee (no. 1195/2014) and conducted according to the guidelines of the Declaration of Helsinki on biomedical research involving human subjects. Written informed consent was obtained from all patients prior to the tests and questionnaires at follow-up.

### Causes and duration of olfactory dysfunction

Table 1 depicts the causes of OD. The time between first symptom of OD and first consultation at our department was 36.2 ± 61.2 months. All three patients with Kallmann’s syndrome could not remember when they first noticed the olfactory impairment. Posttraumatic OD included six patients who slipped and fell on solid ground and three patients with car or bike accidents; the accident mechanism of one patient was not documented.

**Table 1:** Causes of OD and subjective reported olfactory function at follow-up

	Causes (*n*^a^/%)		Improvement (*n*/%)		No change (*n*/%)		Deterioration (*n*/%)	
Postinfectious	29	44.6	14	48.3	11	37.9	2	6.9
Posttraumatic	10	15.4	3	30.0	7	70	0	0
Idiopathic	13	20.0	2	15.4	8	61.5	0	0
Sinonasal	6	9.2	2	33.3	4	66.7	0	0
Congenital	3	4.6	0	0	3	100	0	0
Toxic	3	4.6	1	33.3	1	33.3	1	33.3
Iatrogenic	1	1.5	0	0	1	100	0	0

^a^Number and percentage of the causes for OD and number of patients who stated an improvement, no change or a deterioration of OD; 2 (6.9%) patients with postinfectious OD and 3 (23.1%) patients with idiopathic OD did not answer the question and are therefore not included in the table

### Questionnaires at follow-up

At follow-up, between 2014 and 2016 the VAS for olfactory function was collected and patients were asked if their dysfunction had improved/worsened, or had not changed compared to initial consultation. Additionally, the following questions were asked: “Are there any negative or positive effects due to OD?”, and “Does your OD affect your QoL?”.

The BDI-II consists of 21 statements defining the grade of depressive illness [26–28]. Notably, this questionnaire is not intended to diagnose depression but it assesses the severity of depressive symptoms and can be used as a screening tool within the general population. Patients are asked to pick one statement that defines their state of mind over the last 2 weeks. The possible answers include “I do not feel sad”, “I feel sad”, “I am sad all the time and I cannot snap out of it”, and “I am so sad or unhappy that I cannot stand it”. A maximum score of 3 for each question is summarized; a sum score of 0–8 points indicates no depressive symptoms, 9–13 points minor, 14–19 points mild, 20–28 points moderate and 29–63 points severe depressive symptoms. If more than 10% of the questions were not answered, the total score was rejected. Data from 56 patients could be analyzed.

The WHOQOL-BREF questionnaire (WHOQOL Group, 1998) was developed to assess the QoL in a short form. It consists of 26 questions grouped into 4 domains: physical health, psychological health, social relationship, and environment. Each question is scored according to a 5-point Likert scale. The mean score of all items corresponds to the domain score and is multiplied by 4 in order to compare the scores to the WHOQOL-100. A high score indicates high QoL. More than 20% missing data within each domain leads to a rejection of the score. Complete data from the WHOQOL-BREF were available for 61 patients.

### Olfactory testing at follow-up

At the follow-up examination, olfactory testing was assessed using the pen-like Sniffin’ Sticks test (Burghart Messtechnik GmbH, Wedel, Germany) [24, 29–31]. The odor identification test kit, which includes 16 suprathreshold odors that serve as a screening tool for olfactory disorders, was self-administered [32]. A list of four descriptors for each odor was provided for identification. Due to visual impairment, two patients were provided with assisted olfactory testing [33]. The number of correctly identified odors was counted to obtain the identification score. Normal olfactory function is assumed by ≥13 points. Patients older than 55 years should score at least 12 points to indicate normal function. A total of 34 patients performed a smell identification test at follow-up. Smell identification scores from both clinical contacts were available for 19 patients.

### Statistical analysis

Statistical analysis and graphical visualization were performed using GraphPrism 8.4.2. (GraphPad Software, La Jolla, CA, USA). Data normality was tested using the Shapiro-Wilk test (see supplementary material). To compare scores in an explorative manner, we used paired t‑tests and Mann-Whitney U-tests depending on the distribution. Results of t-tests are presented along with t-statistics [paired-t(degrees of freedom) = t-value]. Correlational analyses were performed using Spearman rank correlation. The alpha level was set at 0.05.

## Results

### Subjective rating of olfactory function

Out of the 65 included patients, 31 (47.7%) patients returned for the initial recommended 6 months follow-up (short-term follow-up) 18.6 weeks after the first clinical contact. Improvement of OD was stated by 11 (16.9%) patients and no change by 20 (30.8%) patients. The residual 34 (52.3%) patients did not show up for a short-term clinical examination.

On study follow-up, the question whether olfactory function improved, worsened, or did not change compared to the first clinical contact was answered as follows: improvement of OD was reported by 33.8% (*n* = 22), whereas 53.8% (*n* = 35) stated no change and 4.6% (*n* = 3) suffered from worsened symptoms (response rate 92% of 65 included patients) compared to the situation at first clinical contact. Table 1 reports the olfactory function at follow-up.

**Fig. 2:**
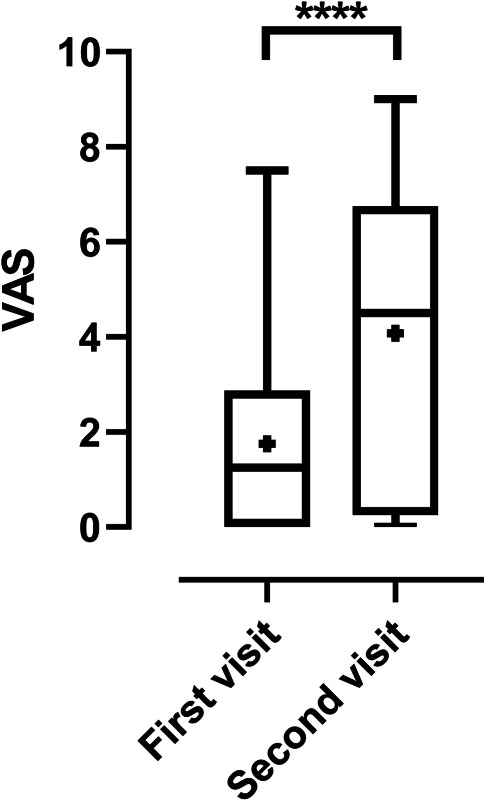
Self-rated smell function at first and follow-up visits. The VAS score at follow-up was significantly higher than the VAS score at the first clinical contact, ****p < 0.001, *n* = 40. Boxes (1st–3rd quartile) represent the middle 50% of data, the inside horizontal lines mark the median, the plus signs mark the mean values and the two horizontal lines mark the whiskers (minimum to maximum)

The VAS ratings from both the first clinical examination and study follow-up were available for 40 patients. At first clinical visit, the median VAS score was 1.25 compared to a mean VAS score of 4.5  at follow-up (U = 469.5, *p* = 0.001; Fig. 2). Median VAS scores at initial clinical visit were significantly different between patients who sent questionnaires only (median = 2.25) and patients who performed a clinical follow-up examination (median = 1.0; U = 148, *p* = 0.041).

### Olfactory testing

The mean ± standard deviation (SD) identification score at the first clinical contact was 6.0 ± 3.0. At follow-up, the mean ± SD identification score was 8.0 ± 4.0. Significantly higher identification scores were found at follow-up [t(18) = 2.51, *p* = 0.021]. Mean ± SD identification scores at first clinical visit were significantly different between patients who sent questionnaires only (8.4 ± 3.9) and patients who underwent a clinical follow-up examination (6.3 ± 3.0; [t(45) = 2.039, *p* = 0.047]).

### General quality of life outcome

**Fig. 3:**
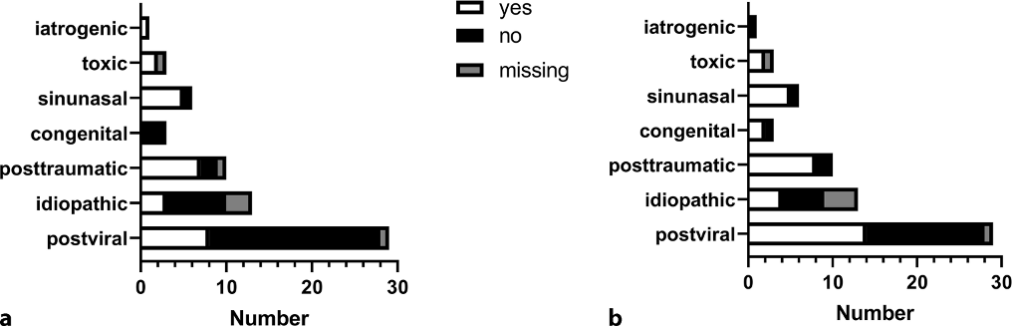
Number of patients who stated an effect of olfactory disorders. **a** Effect on quality of life as yes/no answers for all patients and individual causes. **b** Negative effects reported as yes/no answers for all patients and individual causes. *Black bars* indicate “no”, *white bars* indicate “yes”, and *gray bars* indicate missing answers

Out of 65 patients who were asked whether their olfactory function affected their QoL at follow-up, 40.0% (*n* = 26) reported a reduction and 50.8% (*n* = 33) no effect. Fig. 3 depicts QoL ratings for all patients according to the cause of OD.

Negative effects caused by OD were stated by 53.8% (*n* = 35), whereas 36.9% (*n* = 24) reported no negative effects (6 missing). In contrast, 32.3% (*n* = 21) could see some advantages in their olfactory impairment concerning mostly unpleasant odors (e.g., smell in public transportation, restrooms) and 61.5% (*n* = 40) reported no advantages (6 missing).

#### WHOQOL-BREF

Mean domain scores for all patients and for each cause of OD are shown in Table 2. The score in the physical health domain was highest in iatrogenic OD (78.6), followed by congenital (72.6 ± 13.5) and idiopathic (64.1 ± 15.3) OD and was lowest in toxic OD (46.4 ± 20.2). The highest score in the psychological health domain was observed in patients with congenital OD (81.9 ± 12.0) and lowest in idiopathic OD (57.5 ± 19.9). Scores in the social health domain were highest in patients with iatrogenic (83.3) and congenital OD (80.6 ± 26.8) and lowest in toxic (45.8 ± 5.9) and sinonasal OD (58.3 ± 23.0). The environment domain was scored highest by patients with iatrogenic OD (83.3), whereas the sum scores for other causes of OD were similar.

**Table 2:** WHOQOL-BREF scores of the study population

	Physical health	Psychological health	Social health	Environment
All patients	62.1 ± 15.4	66.7 ± 18.4	62.5 ± 22.0	74.2 ± 15.2
Postinfectious	60.7 ± 17.7	66.8 ± 19.0	61.8 ± 24.0	75.4 ± 16.8
Idiopathic	64.1 ± 15.3	57.5 ± 19.9	59.6 ± 21.2	71.1 ± 17.0
Posttraumatic	62.5 ± 9.4	71.3 ± 13.8	66.7 ± 16.2	73.4 ± 8.6
Congenital	72.6 ± 13.5	81.9 ± 12.0	80.6 ± 26.8	75.0 ± 16.5
Sinonasal	61.9 ± 13.5	66.7 ± 23.3	58.3 ± 23.0	70.3 ± 15.5
Toxic	46.4 ± 20.2	62.5 ± 0.0	45.8 ± 5.9	73.4 ± 15.5
Iatrogenic^a^	78.6	75.0	83.3	96.9

Domain scores as mean ± standard deviation

^a^Iatrogenic group included only one patient

#### Beck’s depression inventory

Table 3 shows data of the BDI-II at follow-up. The highest mean scores were observed in patients with toxic and idiopathic OD (13.8 ± 13.8 and 9.5 ± 10.3, respectively), whereas patients who suffered from congenital (2.0 ± 3.5) and iatrogenic (2.0) OD stated no depressive symptoms.

**Table 3:** BDI-II scores of study population

All patients	8.3 ± 7.4
Postinfectious	8.5 ± 7.4
Idiopathic	9.5 ± 10.3
Posttraumatic	8.3 ± 5.0
Congenital	2.0 ± 3.5
Sinonasal	8.3 ± 6.2
Toxic	13.8 ± 13.8
Iatrogenic^a^	2.0

BDI-II scores as mean ± standard deviation

^a^ Iatrogenic group included only one patient

## Discussion

The present study investigated the subjective long-term outcome of OD after an average of 8.6 years. Compared to the short-term outcome including a 16.9% improvement rate, OD further improved in the long-term in 33.8% of included patients, which was confirmed by increased odor identification scores. It seems that the highest rate of subjective recovery was assessed in patients suffering from postinfectious OD. Similar rates were found by Cavazzana et al., who reported improvement in 46% of anosmic and 35% of hyposmic patients after a shorter follow-up of 1.9 years [34].

Recovery of traumatic OD seems to depend on the trauma severity and the affected area within the brain. Studies examining the relationship between the severity of the trauma and the occurrence of OD showed mixed results [11, 35]. In the current study, subjective improvement was noted in 30% of posttraumatic patients. Most of the current examined patients experienced blunt head trauma with contusions due to falls. Three patients were considered to suffer from severe head trauma as unconsciousness or fracture of the skull base was diagnosed. It seems that improvement rates of OD increases over time; previously published literature reported on recovery rates of 10% after 13.6 months [36], 12.5% after 39.1 months [37], and 35% after 38.1 months [8]. Doty et al. found that 36% improved within 0.5–13 years after the trauma [38]. The same authors stated a relation between trauma severity and hyposmia, whereas no relation was found in anosmic patients; however, the etiology per se does not seem to be predictive for recovery [39]. Thus, the outcome after posttraumatic OD is highly variable depending on the patient cohort. Consequently, the prognosis of OD after head trauma should not be predicted as poor at an early stage of disease.

Only six patients with sinonasal OD took part in this follow-up examination. One reason for this low number is that patients with chronic sinusitis suffer primarily from symptoms other than OD and, therefore, do not present initially at the specialized smell and taste clinic. In sinonasal OD, recovery rates depend on the extent of olfactory dysfunction (anosmia vs. hyposmia) and treatment regimen [40–42], but no further conclusions for this specific group can be drawn from the current study.

Diagnosis of idiopathic OD is difficult and other possible causes should be carefully ruled out [12]. In some cases, idiopathic OD is associated with the development of Parkinson’s disease as a distinct entity of OD [43]. In the current study, recovery in this group of patients was poor and no difference in the VAS score was found between the first contact and follow-up visit.

As expected, all patients with Kallmann’s syndrome reported no change in olfactory function because they had never experienced olfactory sensations. Undoubtedly, this is a special patient group and cannot be compared to patients with other acquired causes of OD; however, little is known about the influence of OD on daily life in this patient group. Despite the inability to detect hazardous situations [14], the QoL of those included was not affected.

At follow-up, roughly half of included patients reported negative effects concerning personal hygiene, detection of spoiled food, fire, or gas. Moreover, insecurity, vitality, and sadness were mentioned by some patients. These findings underline the importance of adequately counselling patients with OD regarding potential hazards [19]. In contrast, some positive effects of OD concerning unpleasant odors in public areas or restrooms were stated.

The WHOQOL-BREF had a lower mean score in the physical health domain than previously reported in anosmic patients [44]. This difference may be explained by an older population in the present study, which would have more comorbidities. In general, WHOQOL-BREF scores decline with age [45]. For the psychological health domain, a similar mean score of 68.3 was assessed by Kollndorfer et al. [44]. The authors concluded that self-esteem and QoL are important risk factors for developing depression in patients with OD. The highest scores in all domains were stated by patients with iatrogenic and congenital OD but due to the small sample size, conclusions could not be sufficiently drawn. Although patients with a congenital cause were aware of the negative effects on daily activities, QoL was not affected based on WHOQOL-BREF. Similarly, domain scores of patients with posttraumatic OD were comparable or slightly higher than the mean scores of all included patients, even though only one third improved over time. Patients with sinonasal OD scored slightly lower than the mean scores of all patients. General QoL in patients with chronic sinusitis does not seem to be strongly affected by OD [46], but when providing olfaction-associated QoL questionnaires, a clear improvement can be observed after treatment for chronic sinusitis or allergic rhinitis [47].

Assessing depressive symptoms at follow-up, the mean BDI-II scores were rather low, indicating no or minor depressive symptoms. The lowest scores were observed for patients with congenital and iatrogenic OD, suggesting no depression. Interestingly, the highest scores were observed for patients with toxic OD, but due to the small sample size, this can only be interpreted with caution. Patients with postinfectious, sinonasal, and posttraumatic OD had similar mean BDI-II scores. Jung et al. reported that postinfectious OD affects a patient’s mood more severely than chronic sinusitis, with mean BDI-II scores of 14.5 and 9.3, respectively [48]. The authors concluded that the sudden onset of OD affects a patient’s mood more severely than a gradual onset, as in chronic sinusitis, which is a very common finding at our smell and taste clinic. Kollndorfer et al., who compared anosmic patients with normal controls, found no difference with respect to BDI scores [44]. In contrast, previous authors reported an association between depressive symptoms and olfactory loss in chronic sinonasal disease [49, 50]. The same group examined the effect of OD in chronic sinusitis and allergic rhinitis on depressive symptoms. Improved BDI scores were achieved after treatment, but initial scores were just above the cut-off for minor depression [47]. In the present investigation, only patients with idiopathic and toxic OD scored in the range that corresponds to minor depression.

Up to now, there is no consensus on which questionnaire best reflects the effect of OD on QoL. In the current study, olfaction non-specific questionnaires were used as depressive symptoms and QoL were not routinely assessed at the first clinical contact in all patients. We assumed that if patients were severely affected by any present OD, a significant reduction of QoL would have been observed at follow-up [47]. Although not asked specifically, many patients reported that they would not have rated their olfactory function as important as long as they had not experienced any OD. This further points out the need for assessing the patient’s QoL in the clinical routine initially and in the long-term, preferably in terms of overall and olfaction-specific QoL. It should be further pointed out that the assessment of mood is another important step in patient counselling, as a small proportion of patients is prone to develop depressive symptoms. Therefore, patients could acquire coping strategies at an early stage before any relevant mood disturbance occurs.

One of the flaws of this study is the relatively poor overall response rate of 20%, which has been also an issue in previous studies and might have been arisen due to the long follow-up time [51, 52]. The following reasons could have also been responsible for the poor follow-up rate: firstly, OD improved and there was no further need for treatment. Secondly, patients adapted to their dysfunction and thirdly, patients were not motivated due to limited treatment options. Unfortunately, almost 20% of all patients could not be traced. It seems that patients with more severe OD based on the VAS and identification scores at initial clinical visit were more likely to present for a clinical follow-up examination. In contrast, it seems that patients with higher VAS and identification test scores at first clinical visit were not willing to take part in the study because OD improved or patients adapted to their dysfunction. Therefore, subjective improvement rates could be underestimated based on current findings.

## Conclusion

This long-term outcome study presents subjective outcome rates in a selected group of patients treated for OD; subjective long-term improvement was reported by approximately one third after an average of 8.9 years with the best prognosis in postinfectious OD. Only a small reduction in QoL according to the WHO-QOL-BREF was observed, and minor depressive symptoms were found only in some patients, suggesting coping over time. Patients with congenital OD were least affected in their QoL. As many patients with OD feel poorly informed about their prognosis, the current findings hopefully aid clinicians in patient counselling. Furthermore, clinicians should be encouraged to assess QoL and mood disturbances routinely in order to initiate acquisition of coping strategies when necessary.

## Caption Electronic Supplementary Material

Results of the Shapiro-Wilk test for normality of distribution
