# Supplementary material for: Long-term impact of olfactory dysfunction on daily life
Source: Wien Klin Wochenschr. 2020 Oct 21;133(19-20):1004–11. doi: 10.1007/s00508-020-01751-5 (PMC8500863; doi:10.1007/s00508-020-01751-5)
Supplement: Supplementary file 1 — Results of the Shapiro-Wilk test for normality of distribution [file 508_2020_1751_MOESM1_ESM.docx]

| **Results of the Shapiro-Wilk test for normality of distribution** | | |
| --- | --- | --- |
| Sample characteristics | Shapiro-Wilk test statistics | p-value |
| VAS rating |  |  |
| First visit | 0.8393 | <0.0001 |
| Second visit | 0.8929 | 0.0012 |
| Questionnaire only | 0.9072 | 0.8451 |
| Clinical follow up | 0.1050 | 0.0006 |
|  |  |  |
| Identification test score |  |  |
| First visit | 0.9595 | 0.5626 |
| Second visit | 0.9636 | 0.6454 |
| Questionnaire only | 0.9630 | 0.5793 |
| Clinical follow up | 0.9485 | 0.2140 |
